# Investigating bronchoalveolar lavage fluid cytology in relation to pathogen identification and consolidation depth in calves

**DOI:** 10.1093/jvimsj/aalaf043

**Published:** 2026-01-21

**Authors:** Justine Clinquart, Thomas Lowie, Stan Jourquin, Jade Bokma, Bart Pardon

**Affiliations:** Department of Internal Medicine, Reproduction and Population Medicine, Ghent University, Salisburylaan 133, Merelbeke-Melle 9820, Belgium; Department of Internal Medicine, Reproduction and Population Medicine, Ghent University, Salisburylaan 133, Merelbeke-Melle 9820, Belgium; Department of Internal Medicine, Reproduction and Population Medicine, Ghent University, Salisburylaan 133, Merelbeke-Melle 9820, Belgium; Department of Internal Medicine, Reproduction and Population Medicine, Ghent University, Salisburylaan 133, Merelbeke-Melle 9820, Belgium; Veterinary Practice Venhei, Geelsebaan 95-97, Kasterlee, 2460, Belgium; Department of Internal Medicine, Reproduction and Population Medicine, Ghent University, Salisburylaan 133, Merelbeke-Melle 9820, Belgium

**Keywords:** pneumonia, bovine respiratory disease, thoracic ultrasonography, airway inflammation, neutrophils, intracellular bacteria

## Abstract

**Background:**

Small volume, non-bronchoscopic bronchoalveolar lavage (nBAL) is used for airway sampling in calves with respiratory tract infection (RTI). However, the usefulness of cytological analysis of this fluid to indicate bacterial RTI is unclear. Disease severity may influence these findings.

**Hypothesis/Objectives:**

Investigate the associations among pathogen groups (none, viral, opportunistic bacterial infection [OB] with or without viral, *Mycoplasmopsis bovis* with or without OB or viral) and cytological findings, and the association between consolidation depth and cytological findings.

**Animals:**

Eighty-seven calves showing at least 1 clinical sign of RTI from herds experiencing an outbreak of respiratory disease.

**Methods:**

Cross-sectional study. Physical examination, thoracic ultrasonography, nBAL, bacteriology, nanopore sequencing, and cytology were performed. Bayesian hierarchical models were used to assess associations.

**Results:**

The probability of belonging to a certain pathogen group was influenced by neutrophil percentage. Viral (median, 75.9%; interquartile range [IQR], 24.0) and *M. bovis* (median, 72.7%; IQR, 21.2) groups showed consistently high neutrophil percentages, whereas variability was higher in the OB group (median, 68.3%; IQR, 31.7%). Intracellular bacteria (≥1% of white blood cells, odds ratio [OR], 12.5; 95% credible interval [CrI], 1.46-142) increased the odds of OB isolation. The probability of different consolidation depths was affected by neutrophil percentage, with deeper lesions showing higher percentages (> 3 cm vs. < 1 cm: OR, 1.06; 95% CrI, 1.01-1.12), and by the presence of mast cells.

**Conclusions and clinical importance:**

The neutrophil percentage alone is likely insufficient to differentiate bacterial from viral infections. Intracellular bacteria might be useful to indicate OB. Evaluation of an optimal threshold could further evaluate this possibility.

## Introduction

The high incidence of respiratory tract infections (RTIs) in young cattle, along with their harmful effects, undermines high welfare standards, optimal economic growth, and minimization of antimicrobial use.^1–6^ To decrease antimicrobial use while maintaining animal welfare, antimicrobial treatment is best reserved for animals suffering from a non-self-limiting bronchopneumonia with bacterial involvement.^7^^,^^8^ Although the diagnosis of bronchopneumonia has been improved by the use of thoracic ultrasonography (TUS),^9^^,^^10^ diagnosis of bacterial involvement requires pathogen identification using respiratory samples. Such samples can be collected by non-bronchoscopic bronchoalveolar lavage (nBAL), a feasible and affordable method for sampling the lower airways. Despite recent developments, laboratory testing for pathogens requires multiple days, and interpretation can be difficult, especially for *Pasteurellaceae.*^11^ Although these bacteria are known to cause severe pneumonia, their presence in healthy calves^12^ complicates the assessment of their role in sampled diseased animals.

In other species, respiratory cytology has been found to indicate the presence or absence of bacterial involvement while awaiting pathogen identification.^13–15^ For example, a study in humans reported a good predictive value of < 50% neutrophils for the absence of bacterial infections^13^ and intracellular bacteria showed good diagnostic value for bacterial involvement in dogs.^14^^,^^16^ In addition, viral infections had lower neutrophil percentages than did bacterial infections in humans.^15^ In calves, direct comparison of cytologic variables in naturally occurring RTI with different involved pathogens has not yet been reported. Thus, cytology potentially could indicate the presence or absence of certain pathogens in anticipation of, or alongside, results of microbiological analysis.

Bronchoalveolar lavage fluid (BALf) cytology is influenced by airway inflammation arising from a complex interaction of cellular and molecular components, either initiated by microorganisms or by noninfectious factors such as air pollutants.^17^ Neutrophils play a key role in the pathogenesis of RTIs. Although they are essential in the protection against microorganisms, they can cause marked tissue damage and contribute to disease severity.^18–21^ The extent of neutrophilic infiltration indeed has been associated with the severity of pathological lesions in bovine respiratory syncytial virus (BRSV)-induced infections in calves.^22^ Subsequently, neutrophil percentages are likely higher, and other cytologic variables, such as the abundance of multinuclear macrophages or epithelial cells, might be altered in calves with severe disease.

Therefore, our primary objective was to explore associations between cytological findings, including neutrophilic proportion, with identified pathogens in nBAL-obtained BALf (nBALf) from calves originating from herds experiencing an outbreak of respiratory disease. The secondary objective was to investigate the associations between the same cytological findings and consolidation depth on lung ultrasonography as a proxy for disease severity.

## Materials and methods

### Animals and study design

A retrospective cross-sectional study was conducted with clinical, ultrasonographical, and microbiological data and cytological preparations of nBALf collected in the winter of 2021-2022 as part of a previous study.^23^ The study protocol was approved by the local ethical committee of the Faculty of Veterinary Medicine, Ghent University, under experimental license number EC2021-079.

For herd selection (dairy, beef, or veal), local veterinarians (*n* = 45) participating in a TUS research project were asked to report respiratory disease outbreaks observed by themselves or their clients via telephone. Herds then were visited within 24 hours by the research team, with treatment postponed until the visit, except in calves with clinical condition that required immediate care. Outbreak determination was based on the veterinarian’s and farmer’s history and clinical observations. An outbreak was defined as ≥ 5 calves displaying at least 1 clinical sign (cough, rectal temperature ≥ 39.4°C, respiratory rate ≥ 44 breaths/min, nasal or eye discharge) within 48 hours in an airspace housing < 33 animals, or ≥ 15% of calves if > 33 were present. The sample size and the relative presence of herds belonging to different production types were limited by financial constraints and the availability of herds reporting an outbreak of respiratory disease in the original study.

### Examination and sampling

Within a herd, the calf inclusion criteria for enrollment included one or more clinical signs associated with respiratory disease (fever, purulent or mucopurulent nasal discharge, ocular discharge, spontaneous or induced cough, increased respiratory rate, depressed mental state, or some combination of these), both pre- and postweaning. This approach was chosen instead of clinical scoring systems to allow inclusion of calves with mild clinical disease. Calves that received antibiotics or noninflammatory drugs within the month before the visit of the research team were excluded. Treatment records before this period were not included. These criteria, and the herd inclusion criteria, aimed at maximizing the inclusion of calves with acute clinical disease according to the objectives of the original study. Up to 7 animals per herd were conveniently selected based on availability.

After selection, clinical scoring, TUS, and nBAL sampling were performed by veterinarians of the research group, who all received consistent training under the supervision of the senior author and had at least 1 year of experience with these techniques. Observations were recorded in Microsoft Excel (Washington, US) using a portable computer device. Clinical scoring, solely intended for description of the study population, was performed following the California clinical scoring system, with a score ≥ 5 considered positive, as described elsewhere.^24^ Lung consolidations were diagnosed by a previously described quick thoracic ultrasound technique (qTUS).^25^ A portable ultrasound unit (KX5200 VET, Kaixin, Bila Tserkva, Ukraine) was used with a linear probe of 5.5-7.5 MHz. Consolidation depth was measured perpendicular to the pleural line using the grid of the ultrasound screen, and the maximal depth was recorded. Collection of nBALf was done using a sterile polytetrafluorethylene catheter, which was advanced through the ventral nasal meatus and passed through the larynx until it was wedged in a bronchus.^26^ One aliquot of 30 mL sterile saline was instilled. If < 9 mL was recovered, a second aliquot of 30 mL was used. Afterward, nBALf was transported on ice after separation in a 1 mL aliquot for cytology in EDTA tubes (1 mL, Vacutest Kima, Azergrande, Italy) and the remaining aliquot for pathogen identification in sterile falcon tubes (Nerbeplus, Winsen, Germany).

### Pathogen identification

Metagenomics, in particular nanopore sequencing (MinION, Oxford Nanopore Technologies, Oxford, UK) was conducted in an external laboratory (PathoSense, Ghent, Belgium) for identification of viral pathogens and *M. bovis* as previously described.^27^^,^^28^ This technique was not used for other bacteria because of a lack of validation for this purpose at the time of sampling. A calf was considered positive for a viral pathogen when bovine coronavirus, bovine respiratory syncytial virus, or parainfluenza 3 was identified. Bovine herpes virus 1 and bovine viral diarrhea virus were not included because of national eradication programs and their subsequent absence in the samples. Influenza D was not included because of its absence in the samples.

Bacterial cultures were used to identify *Pasteurellaceae* and *Trueperella pyogenes*, and *M. bovis. Pasteurellaceae* and *T. pyogenes* were isolated by conventional culture (Columbia blood agar enriched with 5% sheep blood, Oxoid, Hampshire, UK), and selective indicative agars were used for *M. bovis.* Subsequently, samples were incubated in 5% CO_2_-enriched atmosphere at 37°C for 24-47 hours (*Pasteurellaceae* and *T. pyogenes*) or 5-7 days (*M. bovis)*. Identification afterward was achieved using phenotypic characteristics and matrix-assisted laser desorption ionization-time of flight mass spectrometry (MALDI-TOF MS, Brüker Daltonik GmbH, Bremen, Germany).^28^^,^^29^ Calves were considered positive for *M. bovis* after identification of this bacterium by culture or nanopore sequencing. Cultures of other bacteria were recorded using a semiquantitative classification described elsewhere.^29^ Because of their common presence in the microbiome of healthy animals, the results of these bacteria were grouped under the term opportunistic bacteria (OB). Calves were considered positive for OB when a dominant, mixed, or pure culture of *Pasteurella multocida, Mannheimia haemolytica, Histophilus somni, Gallibacterium anatis*, or *T. pyogenes* was recorded. Negative, polymicrobial, or cultures with few colonies were not considered positive, to minimize false-positives resulting from upper airway contamination.

### Cytologic analysis

Cytologic examination consisted of differential counts performed on cytospin preparations and the assessment of additional characteristics. Cytospin preparation occurred within 6 hours after sampling, using cytocentrifugation at 129 relative centrifugal force for 10 minutes (Shandon Scientific, London, UK). Afterward, samples were stained using a rapid staining solution (Hemacolor, Merck KGaA, Darmstadt, Germany). Preparations were blinded from calf and herd information before analysis. Cytologic analysis was performed by the first author, a veterinarian who received specific training in BALf cytology.

Before analysis, sample quality was evaluated with the exclusion of unsuitable samples. Differential counts were performed by microscopic examination of 500 white blood cells, evenly spread on 2 cytospin preparations of the same sample. A 100× oil objective was used (Euromex Iscope, Arnhem, The Netherlands). White blood cells were differentiated into macrophages, neutrophils, lymphocytes, eosinophils, and mast cells. Macrophages were differentiated into foamy (vacuoles throughout the entire cytoplasm), multinuclear (more than 2 nuclei), and normal macrophages. In addition, bronchial epithelial cells, intracellular bacteria (rod or cocci in macrophages or neutrophils), and the presence of giant macrophages (at least 10 nuclei) were noted. The recording of cytological results was facilitated by a custom-made smartphone application (Thunkable, San Francisco, US). In 8 samples where only 1 suitable cytospin preparation was present, 2 separate counts of 250 cells were performed, each starting at the opposite side of the preparation, with overlap not allowed. In 2 samples where < 500 cells were present, the maximal number was used, with a minimum of 380 cells.

### Statistical analysis

Statistical analyses with the calf level as experimental unit were performed using R (version 4.4.1)^30^ and figures were created using “ggridges” and “brms” packages,^31^^,^^32^ and Adobe Illustrator (version 16.0.0, Adobe, San Jose, California). Summary statistics were used for descriptive analysis of demographic and clinical data. To explore potential associations, Bayesian hierarchical categorical models were performed using the “brms” package,^32^ because they allow the inclusion of a random effect (herd) in multinomial models. Calves with missing data were excluded from analyses involving the affected variable.

### Outcome and predictor variables

Outcome factors of interest were ultrasound severity and the identified pathogens, which were each modeled as a multinomial outcome factor in 2 separate models. For classification of the consolidation depth, the qTUS scoring system was used,^33^ creating 4 categories based on the maximum consolidation depth: no consolidation, mild (<1 cm consolidation), moderate (≥1 cm-<3 cm consolidation), and severe (≥3 cm consolidation). Because of the limited sample size, 4 pathogen groups were defined based on clinical relevance. Bacterial involvement was considered indicative of potential benefit from antibiotic treatment, regardless of concurrent viral presence. The detection of *M. bovis* specifically necessitates targeted antibiotic treatment^34^ and additional control measures. Therefore, pathogens were grouped as follows: no identified pathogens, viral pathogens, OB with or without viral pathogens, and *M. bovis* with or without pathogens of the other groups. Predictor variables of interest consisted of neutrophils, eosinophils, mast cells, intracellular bacteria, foamy macrophages, multinuclear macrophages, presence of giant macrophages, and epithelial cells. Neutrophils were examined as a continuous variable. For mast cells, a binary variable (absent-present) was created because of their very low numbers and the less suitable stain for this cell type hindering reliable enumeration.^35^ Because only 3 samples contained > 1% eosinophils, it was determined not to be useful for further analysis in the study. Intracellular bacteria, foamy macrophages, multinuclear macrophages, and epithelial cells were assessed both as continuous and binary variables. Intracellular bacteria counts were determined by absolute number and a binary classification of < 5 versus ≥ 5 (or ≥ 1% of the white blood cells if < 500 cells were available). For foamy macrophages and multinuclear macrophages, the percentage of the total number of macrophages was calculated for continuous variables, and thresholds of ≥ 10% and ≥ 2% were used for the binary variables of foamy and multinuclear macrophages, respectively. Epithelial cells were expressed as a ratio relative to the number of white blood cells, and for the binary variable, a threshold of ≥ 0.25 (1 epithelial cell per 4 white blood cells) was used.

### Variable and model selection

Multiple correlation and Cramer’s *V* (between outcome and predictor) were determined using the “greybox” package.^36^ Variables that showed very low correlation coefficients or Cramer’s *V* < 0.06 with the outcome factor were not included in further analysis of that outcome. The choice between binary and continuous for applicable variables was made based on the best model fit for univariable models, assessed by the leave-one-out information criteria (LOOIC), calculated using the “loo” package.^37^ This measure is based on the predictive accuracy of the model while inherently penalizing overfitting. In brief, the model is repeatedly fitted to the data with 1 observation left out each time, assessing its ability to predict the omitted point. The LOOIC score then aggregates these results, with lower values indicating a better fit. First, a model with all remaining variables was run, and variables where no indication of any effect was present were removed (95% CrI including 1 and very wide at both sides). The remaining model options were compared, and the final model was chosen based on the lowest LOOIC. However, because neutrophils were of specific interest, this variable was fixed in each model.

### Model characteristics

All models were categorical models using the logit link function. Models were run with 4 chains of 1500 iterations with a warm-up of 500 for comparison models and 4000 iterations with a warm-up of 1000 for final models. Weakly informative priors were used (normal [0, 5] for intercepts, normal [0, 3] for estimates, and Student’s *t* [3, 0, 2.5] for standard deviations of the random effect). Model convergence was achieved in each model as assessed by R-hat, effective sample size (ESS), trace plots, autocorrelation plots, and posterior predictive check plots. To assess robustness, sensitivity analyses were performed by consecutively varying the priors of one of the intercepts (normal [0,3]; normal [0,7]), estimates (normal [0,2]; normal [0,5]), and SDs (Student’s *t* [3,0,1]; Student’s *t* [3,0,5]) to narrower and wider priors. Sensitivity analysis was considered robust if results were not substantially altered by these adaptations.

## Results

### Herd and animals

**Figure 1:**
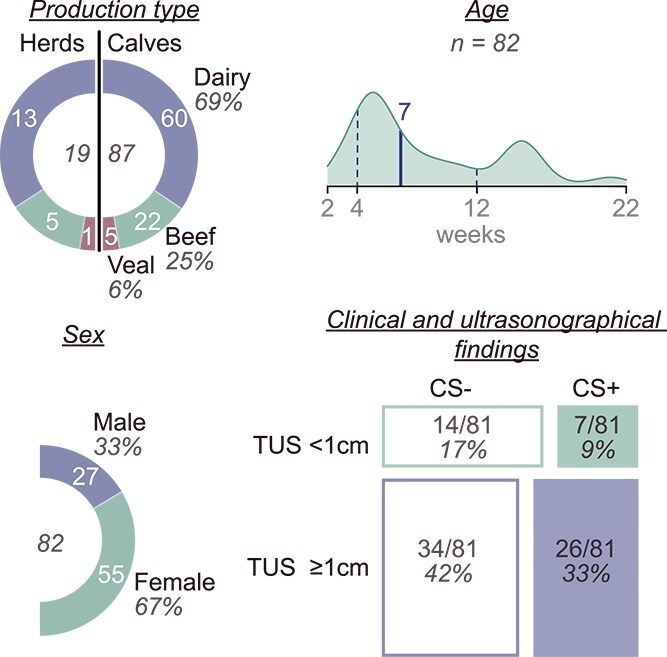
Descriptive data of demographics, clinical and ultrasonographic information of the available information of the 87 studied calves. Calves belonged to 19 herds experiencing an outbreak of respiratory disease. Because of missing information, age, sex, clinical, and ultrasonographic findings were not available for all calves. Production type and sex are represented by doughnut charts with labeling of the total number in the inside of the chart, the absolute numbers in the colored sections, and percentages outside the charts. Age is represented by a density plot with labeling of the minimum, the first quartile, the median, the third quartile, and the maximum from left to right. Clinical and ultrasonographical findings are characterized by a different combination of positive (CS+) and negative (CS−) California score and the presence of a consolidation of ≥ 1 cm on thoracic ultrasonography (TUS).

After the exclusion of 2 samples with inadequate cytological quality, 87 observations from 19 herds (13 dairy, 5 beef, and 1 veal) were suited for the analysis of the outcome of pathogen group, and 86 were suited for analysis of consolidation depth outcome after additional exclusion of 1 sample with missing ultrasonographic data. For 5 observations, demographic and clinical data were not available. Descriptive data of demographic parameters are shown in Figure 1. The median age of the calves was 7 weeks (interquartile range [IQR], 8 weeks; minimum, 2 weeks; maximum, 22 weeks), with a majority of female (67.1%) and dairy calves (69.0%). Because of the selection criteria used based on the presence of at least 1 clinical sign, no truly healthy animals were present. However, 41% of the calves were not positive for the California scoring system. The results of the California scoring system in relation to thoracic ultrasonographic results are shown in Figure 1.

**Figure 2:**
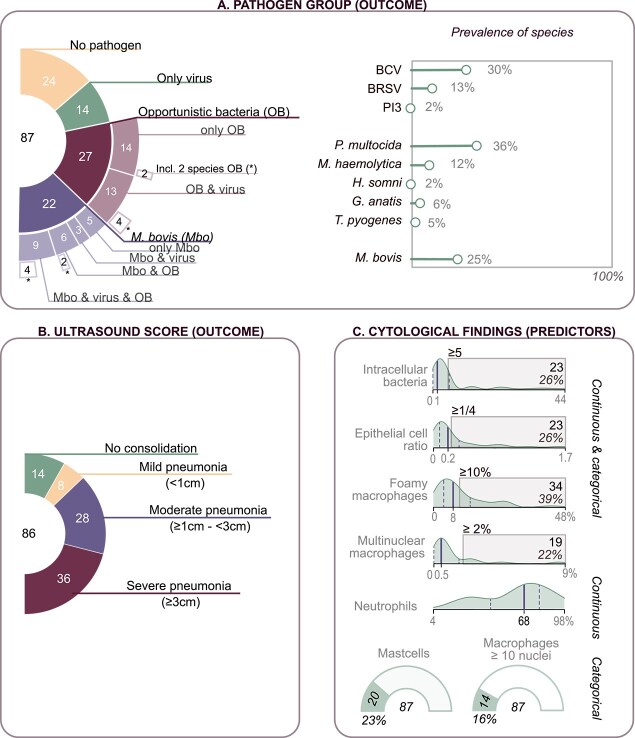
Descriptive data of outcome and predictor variables from studied calves from 19 herds experiencing an outbreak of respiratory disease. Dairy, beef, and veal calves from 2 to 22 weeks and at least one clinical sign of respiratory disease were included. (A) Composition and prevalence of pathogen groups based on bronchoalveolar lavage fluid as the first outcome. On the left, the sunburst chart shows the total number of calves in the inside of the chart, the number of pathogen groups in the inner circle, the number of co-infections in the second circle, and lastly, the number of cases where 2 different opportunistic bacteria were present. The right shows the prevalences of each included pathogen species in all calves (87). (B) Composition of groups based on the qTUS ultrasound score as second outcome (86 calves). (C) Distribution, prevalence, and categorization of cytological findings of bronchoalveolar lavage fluid as predictors (87 calves). Density plots are labeled with minimum, median, and maximum from left to right. Gray rectangles show values considered positive in categorical analysis.

Descriptive information of the analyzed outcomes (pathogen group and ultrasound score) and the predictors (cytological findings) are shown in Figure 2. For the outcome pathogen group, no pathogen, only virus, OB, and *M. bovis* groups consisted of 27.6% (24/87), 16.1% (14/87), 31.0% (27/87), and 25.3% (22/87) of the calves, respectively. Information regarding co-infections and identified pathogens is presented in Figure 2. Co-infections occurred in 34.5% of the sampled calves. No consolidation, mild pneumonia, moderate pneumonia, and severe pneumonia based on the qTUS score were present in 16.3% (14/86), 9.3% (8/86), 32.6% (28/86), and 41.9% (36/86) of the calves, respectively.

### Identified pathogens

**Figure 3:**
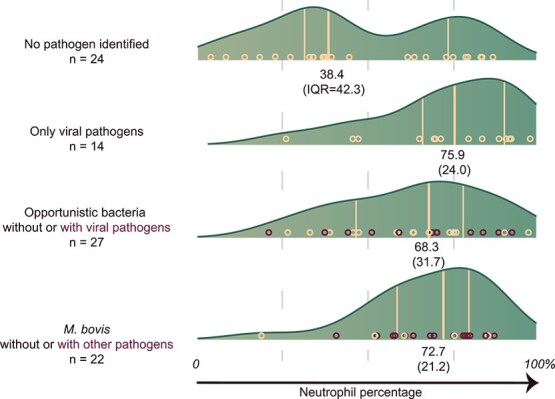
The grouped density plot of neutrophil percentage of bronchoalveolar lavage fluid among different identified pathogen groups. Results of 87 group-housed calves from herds experiencing an outbreak of respiratory disease. Red points represent samples where co-infections were found (opportunistic bacteria and viral pathogen, *M. bovis* with viral pathogen or opportunistic bacteria).

**Figure 4:**
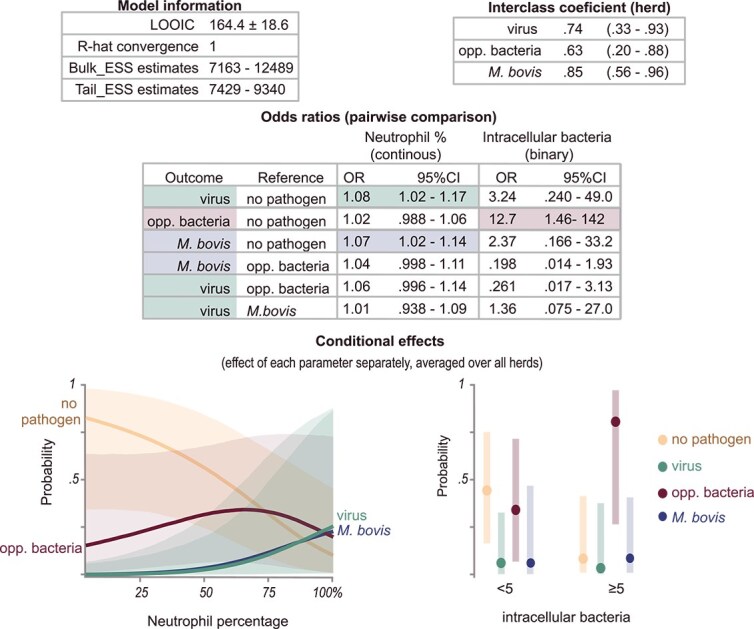
Results of the final Bayesian hierarchical categorical model with the pathogen group as the outcome and the cytological findings as the predictors. Analysis was performed on 87 group-housed calves from herds experiencing an outbreak of respiratory disease. ESS = effective sample size, LOOIC = leave-one-out information criterion. Pathogen group was defined as: Viral pathogen presence of only viral pathogens; Opp. Bacteria = presence of opportunistic bacteria with or without viral pathogens; *M. bovis = M. bovis* with or without pathogens of other groups.

The neutrophil percentages among different pathogen groups based on identified pathogens from nBALf are shown in Figure 3. The final Bayesian regression model contained neutrophil percentage and intracellular bacteria ≥ 5. The model is shown in Figure 4. An increase of 1% neutrophils increased the odds of only a viral pathogen being present by 8% (OR, 1.08; 95% CrI, 1.02-1.17) and the presence of *M. bovis* with or without other pathogens by 7% (OR, 1.07; 95% CrI, 1.02-1.14)*.* Samples with intracellular bacteria ≥ 5 (corresponding to presence in ≥ 1% of white blood cells) had higher odds of OB isolation (OR, 12.7; 95% CrI, 1.46-142). The results for neutrophil percentage were robust among the different sensitivity analyses (see Supplementary materials). For intracellular bacteria, the sensitivity analysis indicated that the choice of prior influences the odds ratio estimates and the upper bound of credible intervals, whereas the lower bounds remained robust under prior specifications. This finding suggests that although the magnitude of the estimated effects is less certain, the direction and presence of the observed associations are robust.

### Consolidation depth

**Figure 5:**
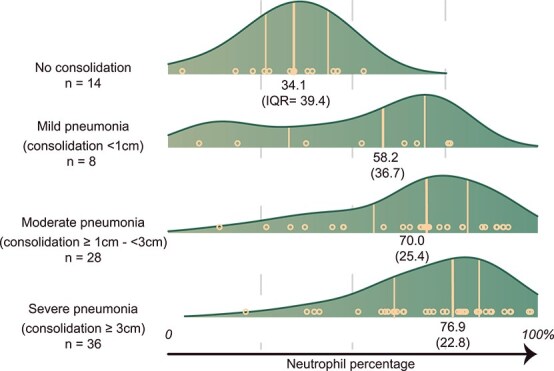
Grouped density plot of neutrophil percentage of bronchoalveolar lavage fluid among different categories of ultrasound lesions. Results of 86 group-housed calves from herds experiencing an outbreak of respiratory disease. The median and IQR are presented underneath each density plot.

**Figure 6:**
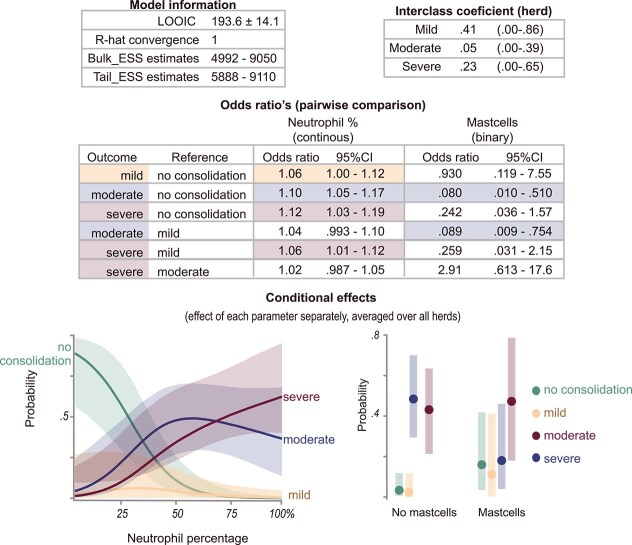
Results of the final Bayesian hierarchical categorical model with ultrasound severity as outcome and cytological findings as predictors. Analysis was performed on 86 group-housed calves from herds experiencing an outbreak of respiratory disease. ESS = effective sample size, LOOIC = leave-one-out information criterion. Ultrasound severity was defined as no consolidation, mild pneumonia (<1 cm consolidation depth), moderate pneumonia (≥1 cm-< 3 cm), and severe pneumonia (<3 cm).

With increasing ultrasound severity assessed by consolidation depth, increasing medians of the neutrophil percentage of nBALf were recorded. Distributions of neutrophil percentage are shown in Figure 5. The final Bayesian regression model for consolidation depth contained neutrophil percentage and the presence of mast cells. This model is shown in Figure 6. An increase in neutrophil percentage was associated with higher odds of all consolidation categories, with the highest odds observed for the severe category compared with no consolidation. In pairwise comparison, increased neutrophil percentages were associated with higher odds of severe consolidation compared with mild (OR, 1.06; 95% CrI, 1.00-1.12). The detection of mast cells decreased the probability of moderate consolidation (OR, 0.080; 95% CrI, 0.010-0.510). The results were robust among the different sensitivity analyses (see Supplementary materials).

## Discussion

Cytological findings of BALf have been proposed in other species as indicators of the absence or presence of bacterial involvement.^13^^,^^16^ Although this finding would be useful in cattle, comparison of cytological variables derived from the commonly performed nBAL among different identified pathogens was not previously reported. In addition, neutrophils play a crucial role in airway inflammation and injury. Therefore, their accumulation is likely influenced by and potentially contributes to disease severity, resulting in altered neutrophil percentages in nBALf.^22^^,^^38^ In the studied herds experiencing an outbreak of respiratory disease, the isolation of OB (with or without concurrent viral pathogen) had a higher probability when intracellular bacteria (≥1% of white blood cells) were found in cytology. Identification of all pathogen groups, albeit with less certainty for OB, had a higher probability when higher neutrophil percentages were found. Nevertheless, probabilities on pathogen groups showed considerable overlap, hindering the distinction among these groups. The probability of having deeper consolidations on TUS increased with increasing neutrophil percentage.

Respiratory samples, like nBAL, are commonly taken for pathogen identification and subsequent antibiotic treatment guidance and preventive strategies in herds experiencing outbreaks of respiratory disease. In contrast to pathogen identification techniques, cytology results can be obtained on the same day as the sampling. Therefore, these results could guide treatment in anticipation of the results of further pathogen identification, or may aid in the interpretation of the importance of the identified pathogens. In our study, a Bayesian hierarchical probability model was developed for the prediction of pathogen groups (no pathogen, virus only, OB with or without virus, *M. bovis* with or without other pathogens). The model with the best fit contained the neutrophil percentage and intracellular bacteria.

Firstly, neutrophil percentage was higher in calves where a pathogen was detected, which also was confirmed for identification of the virus group (OR, 1.08; 95% CrI, 1.02-1.17), for the *M. bovis* group (OR, 1.07; 95% CrI, 1.02-1.14) and with less certainty for OB (OR, 1.02; 95% CrI, 0.988-1.06) in a model accounting for herd effect. This observation is consistent with previous studies that reported associations between neutrophil percentages and *P multocida* and *M. bovis.*^39^^,^^40^ The association however was not consistent for all *Pasteurellaceae*, and the association with *M. bovis* was only found in 1 of the 2 studies.^39^ In one of these studies, the reported neutrophil percentages were lower for the previously mentioned OB compared with our study.^39^ These lower percentages are likely a consequence of the inclusion of epithelial cells in the differential count in that study, as well as the sampling of herds with endemic respiratory disease. The sampled disease stages likely impact neutrophil percentages because neutrophil recruitment is a dynamic response.^41^^,^^42^ It is noteworthy that a substantial number of animals from the no pathogen group in our study still had high neutrophil percentages, and the density plots showed a bimodal distribution. This finding could indicate false negatives, for example, because of poor growth caused by a lower burden in early disease stage or overgrowth of causative bacteria in polymicrobial cultures, especially with fastidious organism such as *H. somni*. In addition, noninfectious airway inflammation also could cause an increase in neutrophils, with little information on the magnitude of the increase,^17^ and requires more complex diagnostic testing.

Differentiation of pathogens is essential to serve as an early indicator for specific pathogen groups. In our study, some differences were found among the studied groups, but large overlap prevented clear differentiation. The Bayesian model suggested a higher, albeit less certain, probability of isolating the virus group (OR, 1.06; 95% CrI, 0.998-1.14) and *M. bovis* (OR, 1.04; 95% CrI, 0.998-1.11) group compared with the OB group. Furthermore, the probability for both groups seemed to be highest from 75% onward. To summarize, neutrophil percentages of the virus and *M. bovis* group were more consistently high compared with the OB group, which showed considerable variability. This observation is in contrast to findings in human medicine, where consistent neutrophilia (>50%) was seen in bacterial infections, whereas viral infections exhibited a wide range of neutrophil percentages.^13^^,^^15^^,^^43^ Firstly, the higher neutrophil percentage seen with viral pathogens in our study may be a consequence of the bronchoscopic technique used, and of the different involved pathogens and their prevalence. In addition, neutrophilic recruitment, a crucial component of the inflammatory process, may differ between humans and calves.

For OB, the substantial presence of low neutrophil percentages hindered its effectiveness as a predictor of the absence of OB. These low percentages fell within previously suggested normal reference values for the technique used.^44^ They were present both for sole OB identification and mixed OB and viral identification. These results could be a consequence of nBAL, difficulties in the interpretation of culture results, variability in host immunity response, peripheral leukopenia, and variability in sampled disease stages. The blind nature of nBAL collection can result in sampling of non-affected lung parts. Particularly in bacterial infections, where mostly the cranial parts are affected, sampling of non-affected lung parts is common.^26^ In addition, interpretation of culture results, especially for OB, is difficult because the identification of bacteria that only colonize the respiratory tract without causing any harm or contamination with bacteria present in the nasopharynx cannot be excluded.^12^^,^^45^ Furthermore, variability in the development of disease after respiratory infection has been attributed to differences in host responses to microorganisms.^21^^,^^46^ Because neutrophils are key mediators of airway inflammation,^47^ individual variability in neutrophil increases is likely. Lastly, the *M. bovis* group showed a similar neutrophil percentage to the viral group. The interpretation of the *M. bovis* group is less straightforward, because *M. bovis* was seldom detected as the sole pathogen and could not be studied separately. Samples in which only *M. bovis* was detected (*n* = 5) occurred over a wide range of neutrophil percentages (18.9%-85.7%). Altogether, the findings of our study suggest that neutrophil percentage alone cannot serve as a pathogen predictor.

Intracellular bacteria, however, also were associated with an increased probability of belonging to the OB group, particularly when they were present in ≥ 1% of the white blood cells. This association was a robust finding in our analyses, suggesting its potential as a possible predictor. This observation aligns with findings in human and canine medicine, where intracellular bacteria have been reported as a good indicator of bacterial involvement.^16^^,^^48–50^ However, sensitivity analysis of our study identified uncertainty regarding the magnitude of this association, and this finding was present in only a relatively small number of calves. Although this result limits conclusions about its predictive value, evaluating diagnostic accuracy was beyond the scope of the study and should be assessed in future research. In addition, establishing an optimal threshold to predict or support OB involvement would be valuable, because the current threshold was chosen arbitrarily because of the lack of prior data. Furthermore, the use of Gram-staining also could be evaluated.

Although the aforementioned pathogens stimulate the immune system and initiate an accumulation of neutrophils, not all calves infected with virulent pathogens develop lung lesions. The host’s ability to regulate the immune response was hypothesized to contribute to this phenomenon.^21^ Neutrophils are key mediators in this immune response. Although they produce molecules such as reactive oxygen species, and numerous other molecules as a defense reaction, these mechanisms also cause extensive collateral tissue damage.^18^^,^^20^^,^^38^^,^^41^ Furthermore, excessive and prolonged neutrophil accumulation has been reported in the development of severe pneumonia in several animal models for pneumonia in humans and BRSV in calves.^38^^,^^41^ As a consequence, neutrophil percentages are likely directly related to disease severity. In our study of herds experiencing an outbreak of respiratory disease, a positive relationship was seen between neutrophil percentage and lung consolidation depth. Calves with higher neutrophil percentages had a higher probability of having lung consolidation on TUS, especially consolidations ≥ 1 cm. A few calves, however, had mild consolidations (<1 cm), warranting cautious interpretation. Higher neutrophil percentages additionally were associated with a higher probability of severe lung consolidations (≥3 cm) compared with mild consolidation (OR, 1.06; 95% CrI, 1.01-1.12). Although the probability of severe and moderate (≥1 cm-<3 cm) consolidations was similar in the approximate range of 50%-75%, the probability of severe consolidations increased from 75% on, whereas these decreased for moderate consolidation (conditional plot, Figure 6).

These findings may be a consequence of the previously stated pivotal role of neutrophils in disease development. Furthermore, they are in agreement with a previous study where an association was found between consolidations > 1 cm and neutrophil percentage.^39^ However, no associations between consolidation depth > 3 cm and 6 cm were found in the same study. Although this observation may be a consequence of a different statistical approach, the endemic nature of the sampled calves and variability in housing factors also could have contributed. Firstly, because herds with endemic respiratory problems were sampled, more calves may already be in a resolution phase or could have permanent tissue alterations such as bronchiolitis obliterans, possibly resulting in abnormal findings on TUS.^21^^,^^41^^,^^51^ Secondly, variability in housing factors might have resulted in the variable presence of noninfectious airway inflammation, subsequently influencing neutrophil counts.^17^ Nevertheless, the influence of disease severity on neutrophilic percentage should be considered in the cytologic interpretation of nBALf. Furthermore, the assessment of neutrophil percentage, along with other inflammatory markers, in future research could further elucidate the role of neutrophils in the development of severe RTI. If neutrophils themselves indeed contribute to disease severity as previously suggested,^20^^,^^38^ neutrophil percentage also might serve to identify animals that could benefit from immunomodulatory treatment or serve as a prognostic indicator.

Besides neutrophilic percentage, mast cells also influenced the probability of certain lung consolidation depths in our study. The presence of mast cells decreased the odds of moderate lung consolidations compared with no consolidations and mild consolidations. This finding is in agreement with an experimental study on *M. haemolytica*, where the number of mast cells was decreased in affected lung parts.^52^ This finding might suggest mast cell degranulation in acute disease stages, as previously suggested,^52^ or a relative decrease because of an increase in other cell types. However, results should be interpreted with caution because the staining method used was not reliable for mast cell detection, resulting in an underestimation.^35^

Our study had some limitations. Because of the small sample size, it was necessary to group pathogens and it was impossible to include potential confounders such as age, production type, sex, or recovered volume of nBAL. As a consequence, pathogen-specific effects or the presence of confounders could not be assessed. In addition, the sample size precludes conclusions on the absence of the examined associations. Furthermore, the upper airway passage of nBAL could have resulted in contamination of some samples by upper airway commensals. This possibility is likely minimal because no significant association could be found between polymicrobial nBAL samples and polymicrobial deep nasal swabs from the same calves.^53^ To further minimize this possibility, polymicrobial cultures and cultures with poor growth were not considered positive. In addition to the classic *Pasteurellaceae* (*P. multocida, M. haemolytica*, and *H. somni*), less common bacteria also were considered as OB (*G. anatis* and *T. pyogenes*). Although their contribution to RTI was reported,^54^^,^^55^ their role is less substantiated. Notably, they were only isolated in a minority of the cases, and commonly a concurrent classic *Pasteurellaceae* was present. The inclusion criteria of calves with clinical signs of herds experiencing outbreaks were used to maximize the inclusion of acute clinical disease. However, the presence of calves with chronic or recurrent disease cannot be excluded. In addition, the use of these criteria likely led to an overestimation of the prevalence of pathogen detection, consolidation, and cytological variables. Consequently, the results are likely different in distinct disease dynamics at the herd level and in calves with true subclinical disease. Although including healthy control animals would have been valuable, they were excluded because of the likely increased pathogen circulation in affected herds during the ongoing outbreak. This factor could influence pathogen detection even in healthy animals. In addition, challenges in reliably diagnosing truly healthy calves, especially during a respiratory disease outbreak, and financial constraints supported this decision. Also, TUS was performed by multiple veterinarians, which would likely have resulted in inter-rater variability. However, all veterinarians had substantial experience and underwent extensive training in the technique used, which has been shown to improve the inter-rater agreement.^56^ Lastly, in 2 samples, only 380 and 480 white blood cells could be counted. The impact of these samples was likely minimal because neutrophil percentage after a 300 white blood cell count showed good agreement with a 500 white blood cell count.^57^

In conclusion, the neutrophil percentage, as assessed using a small volume nBAL, likely cannot distinguish different pathogens identified in calves from herds experiencing an outbreak of respiratory disease. However, intracellular bacteria might be useful to indicate or support OB diagnosis. The neutrophil percentage increased with increasing consolidation depth, which might be a result of a possible contribution of neutrophils themselves to disease severity. These findings should be considered in the interpretation of nBAL cytology.

## Supplementary Material

aalaf043_Supplemental_materials_CP

## Off-label antimicrobial declaration

The authors declare no off-label use of antimicrobials.

## Institutional animal care and use committee or other approval declaration

The study was conducted in compliance with the Ghent University rules of animal experiments with the approval of the university’s animal experiment ethics committee (EC2021-079).

## Abbreviations

BALf: bronchoalveolar lavage fluid
BCV: bovine coronavirus
BRSV: bovine respiratory syncytial virus
OB: opportunistic bacteria
nBAL: non-bronchoscopic bronchoalveolar lavage
nBALf: non-bronchoscopic obtained bronchoalveolar lavage fluid
TUS: thoracic ultrasonography
qTUS: quick thoracic ultrasound
