# Supplementary material for: Investigating bronchoalveolar lavage fluid cytology in relation to pathogen identification and consolidation depth in calves
Source: J Vet Intern Med. 2026 Jan 21;40(1):aalaf043. doi: 10.1093/jvimsj/aalaf043 (PMC12881945; doi:10.1093/jvimsj/aalaf043)
Supplement: aalaf043_Supplemental_materials_CP [file aalaf043_supplemental_materials_cp.docx]

## Supplemental materials from Differentiating airway inflammation in calves based on cluster analysis of bronchoalveolar lavage fluid cytology

Abbreviations: SD= standard deviation, Ref. = reference, OR = Odds ratio, L 95% CrI = Lower bound 95% credible interval, H 95% CrI = Higher bound 95% CrI , No Path. = No pathogen, OB = opportunistic bacteria, *M. bovis* = *Mycoplasmopsis bovis,* No cons. = No consolidation.

## Sensitivity analysis outcome pathogen group

#### Priors intercept

| Prior for | Prior | | Influence | | | | |
| --- | --- | --- | --- | --- | --- | --- | --- |
| Intercept | Normal (0,**3**) | | Probability of each group outcome | | | | |
| Estimates | Normal (0,3) | |  |  |  |  |  |
| SD | Student- t (3,0,2.5) | |  |  |  |  |  |
|  | | **Neutrophil percentages** | | | **Intracellular bacteria** | | |
| Outcome | **Ref.** | OR | L 95%CrI | H 95%CrI | OR | L 95%CrI | H 95%CrI |
| Virus | No Path. | 1.08 | 1.03 | 1.17 | 3.40 | .250 | 51.6 |
| OB | No Path. | 1.02 | .988 | 1.06 | 12.5 | 1.45 | 149 |
| *M. bovis* | No Path. | 1.07 | 1.02 | 1.14 | 2.53 | .181 | 36.6 |
| *M. bovis* | OB | 1.05 | .997 | 1.11 | .211 | .015 | 2.00 |
| Virus | OB | 1.01 | .936 | 1.09 | .276 | .020 | 3.09 |
| Virus | *M. bovis* | 1.05 | .993 | 1.13 | 1.31 | .079 | 24.5 |
| Prior for | **Prior** | | **Influence** | | | | |
| Intercept | Normal (0,**7**) | | Probability of each group outcome | | | | |
| Estimates | Normal (0,3) | |  |  |  |  |  |
| SD | Student- t (3,0,2.5) | |  |  |  |  |  |
|  | | **Neutrophil percentages** | | | **Intracellular bacteria** | | |
| Outcome | **Ref.** | OR | L 95%CrI | H 95%CrI | OR | L 95%CrI | H 95%CrI |
| Virus | No Path. | 1.08 | 1.02 | 1.17 | 3.25 | .200 | 51.6 |
| OB | No Path. | 1.02 | .987 | 1.06 | 12.9 | 1.49 | 149 |
| *M. bovis* | No Path. | 1.08 | 1.02 | 1.14 | 2.34 | .152 | 33.1 |
| *M. bovis* | OB | 1.05 | .998 | 1.11 | .190 | .013 | 1.80 |
| Virus | OB | 1.06 | .995 | 1.14 | .265 | .017 | 3.13 |
| Virus | *M. bovis* | 1.01 | .935 | 1.10 | 1.38 | .070 | 29.4 |

#### Priors estimates

| Prior for | Prior | | Influence | | | | |
| --- | --- | --- | --- | --- | --- | --- | --- |
| Intercept | Normal (0,5) | | Odds ratio | | | | |
| Estimates | Normal (0,**2**) | |  |  |  |  |  |
| SD | Student- t (3,0,2.5) | |  |  |  |  |  |
|  | | **Neutrophil percentages** | | | **Intracellular bacteria** | | |
| Outcome | **Ref.** | OR | L 95%CrI | H 95%CrI | OR | L 95%CrI | H 95%CrI |
| Virus | No Path. | 1.08 | 1.02 | 1.17 | 2.05 | .197 | 21.0 |
| *OB* | No Path. | 1.03 | .989 | 1.07 | 7.44 | 1.21 | 52.2 |
| *M. bovis* | No Path. | 1.07 | 1.02 | 1.14 | 1.58 | .162 | 14.7 |
| *M. bovis* | OB | 1.05 | .998 | 1.11 | .218 | 0.02 | 1.96 |
| Virus | OB | 1.05 | .996 | 1.14 | .283 | .024 | 2.88 |
| Virus | *M. bovis* | 1.01 | .939 | 1.09 | 1.29 | .087 | 19.1 |
| Prior for | **Prior** | | **Influence** | | | | |
| Intercept | Normal (0,5) | | Odds ratio | | | | |
| Estimates | Normal (0,**5**) | |  |  |  |  |  |
| SD | Student- t (3,0,2.5) | |  |  |  |  |  |
|  | | **Neutrophil percentages** | | | **Intracellular bacteria** | | |
| Outcome | **Ref.** | OR | L 95%CrI | H 95%CrI | OR | L 95%CrI | H 95%CrI |
| Virus | No Path. | 1.08 | 1.02 | 1.17 | 5.65 | .274 | 152 |
| OB | No Path. | 1.02 | .986 | 1.06 | 22.2 | 1.80 | 477 |
| *M. bovis* | No Path. | 1.07 | 1.02 | 1.14 | 4.07 | .187 | 103 |
| *M. bovis* | OB | 1.05 | .998 | 1.11 | 0.19 | .013 | 1.99 |
| Virus | OB | 1.06 | .995 | 1.14 | .263 | .014 | 3.51 |
| Virus | *M. bovis* | 1.01 | .936 | 1.09 | 1.38 | .064 | 32.6 |

#### Priors group level/ random effect standard deviation

| Prior for | Prior | | Influence | | | | |
| --- | --- | --- | --- | --- | --- | --- | --- |
| Intercept | Normal (0,5) | | Standard deviation of random effect (herd) | | | | |
| Estimates | Normal (0,3) | |  |  |  |  |  |
| SD | Student- t (3,0,1) | |  |  |  |  |  |
|  | | **Neutrophil percentages** | | | **Intracellular bacteria** | | |
| Outcome | **Ref.** | OR | L 95%CrI | H 95%CrI | OR | L 95%CrI | H 95%CrI |
| Virus | No Path. | 1.08 | 1.02 | 1.15 | 3.29 | .259 | 44.3 |
| OB | No Path. | 1.02 | .989 | 1.06 | 10.8 | 1.36 | 118 |
| *M. bovis* | No Path. | 1.07 | 1.02 | 1.13 | 2.56 | .182 | 34.1 |
| *M. bovis* | OB | 1.04 | .996 | 1.10 | .252 | .019 | 2.22 |
| Virus | OB | 1.05 | .994 | 1.13 | .615 | .026 | 3.22 |
| Virus | *M. bovis* | 1.00 | .939 | 1.08 | 1.26 | .080 | 23.1 |

| Prior for | Prior | | Influence | | | | |
| --- | --- | --- | --- | --- | --- | --- | --- |
| Intercept | Normal (0,5) | | Standard deviation of random effect (herd) | | | | |
| Estimates | Normal (0,3) | |  |  |  |  |  |
| SD | Student- t (3,0,**5**) | |  |  |  |  |  |
|  | | **Neutrophil percentages** | | | **Intracellular bacteria** | | |
| Outcome | **Ref.** | OR | L 95%CrI | H 95%CrI | OR | L 95%CrI | H 95%CrI |
| Virus | No Path. | 1.09 | 1.02 | 1.18 | 3.28 | .194 | 57.2 |
| OB | No Path. | 1.03 | .987 | 1.07 | 14.1 | 1.51 | 162 |
| *M. bovis* | No Path. | 1.08 | 1.02 | 1.15 | 2.32 | .149 | 36.4 |
| *M. bovis* | OB | 1.05 | 1.00 | 1.12 | .172 | .011 | 1.80 |
| Virus | OB | 1.06 | .996 | 1.15 | .244 | .015 | 3.28 |
| Virus | *M. bovis* | 1.01 | .935 | 1.1 | 1.4 | .064 | 32.7 |

## Sensitivity analysis outcome ultrasound severity

#### Priors intercept

| Prior for | Prior | | Influence | | | | |
| --- | --- | --- | --- | --- | --- | --- | --- |
| Intercept | Normal (0,**3**) | | Probability of each group outcome | | | | |
| Estimates | Normal (0,3) | |  |  |  |  |  |
| SD | Student- t (3,0,2.5) | |  |  |  |  |  |
|  | | **Neutrophil percentages** | | | **Mast cells** | | |
| Outcome | **Ref.** | OR | L 95%CrI | H 95%CrI | OR | L 95%CrI | H 95%CrI |
| Mild | No cons. | 1.06 | 1.00 | 1.12 | .947 | .123 | 7.25 |
| Moderate | No cons. | 1.10 | 1.05 | 1.16 | .085 | .012 | .514 |
| Severe | No cons. | 1.12 | 1.07 | 1.19 | .256 | .042 | 1.58 |
| Moderate | Mild | 1.04 | .993 | 1.01 | .091 | .009 | .743 |
| Severe | Mild | 1.06 | 1.01 | 1.12 | .269 | .032 | 2.19 |
| Severe | Moderate | 1.02 | .986 | 1.05 | 2.93 | .591 | 17.6 |
| Prior for | **Prior** | | **Influence** | | | | |
| Intercept | Normal (0,**7**) | | Probability of each group outcome | | | | |
| Estimates | Normal (0,3) | |  |  |  |  |  |
| SD | Student- t (3,0,2.5) | |  |  |  |  |  |
|  | | **Neutrophil percentages** | | | **Mast cells** | | |
| Outcome | **Ref.** | OR | L 95%CrI | H 95%CrI | OR | L 95%CrI | H 95%CrI |
| Mild | No cons. | 1.06 | 1.00 | 1.13 | .912 | 0.11 | 7.25 |
| Moderate | No cons. | 1.11 | 1.05 | 1.17 | .080 | .011 | .535 |
| Severe | No cons. | 1.13 | 1.07 | 1.19 | .238 | .037 | 1.60 |
| Moderate | Mild | 1.04 | .995 | 1.01 | .091 | .008 | .766 |
| Severe | Mild | 1.06 | 1.01 | 1.12 | .263 | .031 | 2.10 |
| Severe | Moderate | 1.02 | .986 | 1.05 | 2.84 | .603 | 18.1 |

#### Priors estimates

| Prior for | Prior | | Influence | | | | |
| --- | --- | --- | --- | --- | --- | --- | --- |
| Intercept | Normal (0,5) | | Odds ratio | | | | |
| Estimates | Normal (0,**2**) | |  |  |  |  |  |
| SD | Student- t (3,0,2.5) | |  |  |  |  |  |
|  | | **Neutrophil percentages** | | | **Mast cells** | | |
| Outcome | **Ref.** | OR | L 95%CrI | H 95%CrI | OR | L 95%CrI | H 95%CrI |
| Mild | No cons. | 1.06 | 1.00 | 1.12 | 1.10 | .176 | 6.91 |
| Moderate | No cons. | 1.10 | 1.05 | 1.16 | .120 | .020 | .627 |
| Severe | No cons. | 1.12 | 1.03 | 1.18 | .328 | .062 | 1.75 |
| Moderate | Mild | 1.04 | .994 | 1.09 | .110 | .013 | .791 |
| Severe | Mild | 1.06 | 1.01 | 1.12 | .300 | .039 | 2.17 |
| Severe | Moderate | 1.02 | .988 | 1.05 | 2.67 | .610 | 15.4 |
| Prior for | Prior | | Influence | | | | |
| Intercept | Normal (0,5) | | Odds ratio | | | | |
| Estimates | Normal (0,**5**) | |  |  |  |  |  |
| SD | Student- t (3,0,2.5) | |  |  |  |  |  |
|  | | **Neutrophil percentages** | | | **Mast cells** | | |
| Outcome | **Ref.** | OR | L 95%CrI | H 95%CrI | OR | L 95%CrI | H 95%CrI |
| Mild | No cons. | 1.06 | 1.00 | 1.13 | .795 | .080 | 7.07 |
| Moderate | No cons. | 1.11 | 1.05 | 1.17 | .062 | .007 | .478 |
| Severe | No cons. | 1.13 | 1.07 | 1.20 | .191 | .022 | 1.39 |
| Moderate | Mild | 1.04 | .994 | 1.10 | .080 | .008 | .695 |
| Severe | Mild | 1.06 | 1.01 | 1.12 | .243 | .025 | 2.20 |
| Severe | Moderate | 1.02 | .987 | 1.05 | 3.00 | .593 | 19.6 |

#### Priors group level/ random effect standard deviation

| Prior for | Prior | | Influence | | | | |
| --- | --- | --- | --- | --- | --- | --- | --- |
| Intercept | Normal (0,5) | | Standard deviation of random effect (herd) | | | | |
| Estimates | Normal (0,3) | |  |  |  |  |  |
| SD | Student- t (3,0,**1**) | |  |  |  |  |  |
|  | | **Neutrophil percentages** | | | **Mast cells** | | |
| Outcome | **Ref.** | OR | L 95%CrI | H 95%CrI | OR | L 95%CrI | H 95%CrI |
| Mild | No cons. | 1.06 | 1.00 | 1.12 | 0.938 | 0.127 | 7.06 |
| Moderate | No cons. | 1.10 | 1.05 | 1.16 | 0.084 | 0.01 | 0.528 |
| Severe | No cons. | 1.12 | 1.07 | 1.19 | 0.236 | 0.036 | 1.41 |
| Moderate | Mild | 1.04 | .997 | 1.1 | 0.092 | 0.001 | 0.711 |
| Severe | Mild | 1.06 | 1.01 | 1.12 | 0.254 | 0.033 | 1.83 |
| Severe | Moderate | 1.01 | .988 | 1.05 | 2.73 | 0.577 | 15.4 |
| Prior for | **Prior** | | **Influence** | | | | |
| Intercept | Normal (0,5) | | Standard deviation of random effect (herd) | | | | |
| Estimates | Normal (0,3) | |  |  |  |  |  |
| SD | Student- t (3,0,**5**) | |  |  |  |  |  |
|  | | **Neutrophil percentages** | | | **Mast cells** | | |
| Outcome | **Ref.** | OR | L 95%CrI | H 95%CrI | OR | L 95%CrI | H 95%CrI |
| Mild | No cons. | 1.06 | 1.00 | 1.13 | .901 | .110 | 7.48 |
| Moderate | No cons. | 1.10 | 1.05 | 1.17 | .079 | .011 | .498 |
| Severe | No cons. | 1.13 | 1.07 | 1.20 | .240 | .036 | 1.58 |
| Moderate | Mild | 1.04 | .991 | 1.10 | .089 | .009 | .773 |
| Severe | Mild | 1.06 | 1.01 | 1.12 | .268 | .030 | 2.26 |
| Severe | Moderate | 1.01 | .988 | 1.05 | 2.92 | .626 | 18.3 |
